# Safe surgery checklist: evaluation in a neotropical region

**DOI:** 10.1590/0100-6991e-20202710

**Published:** 2021-03-31

**Authors:** GIULENA ROSA LEITE, MARLENE ANDRADE MARTINS, LUDMILA GREGO MAIA, MARCO TÚLIO ANTONIO GARCIA-ZAPATA

**Affiliations:** 1 - Universidade Federal de Goiás, Programa de Pós-Graduação em Ciências da Saúde da Faculdade de Medicina - Goiânia - GO - Brasil; 2 - Universidade Federal de Jataí, Curso de Enfermagem - Jataí - GO - Brasil

**Keywords:** Checklist, Patient Safety, Surgical Procedures Operative, Lista de Checagem, Segurança do Paciente, Procedimentos Cirúrgicos Operatórios

## Abstract

**Objective:**

assess patient responses and associated factors of items on a safe surgery checklist, and identify use before and after protocol implementation from the records.

**Methods:**

a cohort study conducted from 2014 to 2016 with 397 individuals in stage I and 257 in stage II, 12 months after implementation, totaling 654 patients. Data were obtained in structured interviews. In parallel, 450 checklist assessments were performed in medical records from public health institutions in the Southwest II Health Region of Goiás state, Brazil.

**Results:**

six items from the checklist were evaluated and all of these exhibited differences (p < 0.000). Of the medical records analyzed, 69.9% contained the checklist in stage I and 96.5% in stage II, with better data completeness. In stage II, after training, the checklist was associated with surgery (OR; 1.38; IC95%: 1.25-1.51; p < 0.000), medium-sized hospital (OR; 1.11; CI95%; 1.0-1.17; p < 0.001), male gender (OR; 1.07; CI95%; 1.0-1.14; p < 0.010), type of surgery (OR; 1.7; CI95%: 1.07-1.14; p < 0.014) and antibiotic prophylaxis 30 to 60 min after incision (OR; 1.10; CI95%: 1.04-1.17; p < 0.000) and 30 to 60 min after surgery (OR; 1.23; CI95%: 1.04-1.45; p = 0.015).

**Conclusions:**

the implementation strategy of the safe surgery checklist in small and medium-sized healthcare institutions was relevant and associated with better responses based on patient, data availability and completeness of the data.

## RESUMO

**Objetivo:**

avaliar as respostas de pacientes e fatores associados quanto a itens do checklist de cirurgia segura. Identificar o uso antes e após a implantação de um protocolo a partir dos registros.

**Métodos:**

estudo de coorte realizado entre 2014-2016 com 397 pessoas na etapa I e 257 na etapa II, 12 meses depois da implementação, totalizando 654 pacientes. Os dados foram obtidos mediante entrevista estruturada. Paralelamente, realizaram-se 450 avaliações de checklist em prontuários de instituições públicas de saúde, da Região de Saúde Sudoeste II, Goiás.

**Resultados:**

seis itens foram avaliados da lista, e todos apresentaram diferenças (p < 0,000). Em 69,9% dos prontuários continham o checklist na etapa I, e, após a capacitação, foi identificado em 96,5% dos registros, tendo melhor completude. O checklist, foi associado à cirurgia realizada na segunda etapa, depois da ação educativa, (RP; 1,38; IC95%: 1,25-1,51; p < 0,000), ao hospital de médio porte, (RP; 1,11; IC95%; 1,0-1,17; p < 0,001); sexo masculino (RP; 1,07; IC95%; 1,0-1,14; p < 0,010), tipo de cirurgia, (RP; 1,7; IC95%: 1,07-1,14; p < 0,014) e antibioticoprofilaxia de 30 a 60 min após a incisão (RP; 1,10; IC95%: 1,04-1,17; p < 0,000) e 30 a 60 min após o término da cirurgia (RP; 1,23; IC95%: 1,04-1,45; p = 0,015).

**Conclusões:**

a estratégia de implantação do checklist de cirurgia segura nas instituições de pequeno e médio foram relevantes e associadas a uma melhoria a partir das respostas dos pacientes, na disponibilização e maior completude dos dados.

## INTRODUCTION

Patient safety during surgery in a hospital setting is relevant and requires appropriate management from professionals to reduce the adverse events related to surgical complications^1^ ^,^ ^2^. The surgical treatment brings essential benefits to patients and, according to the World Health Organization (WHO), many procedures are performed in different scenarios, but they must follow principles of safety and quality. In this perspective, WHO launched the Safe Surgery Saves Lives Program, a resource that can contribute to reducing harm and that reports using the safe surgery checklist, applied in several countries^3^ ^,^ ^4^. Thus, in Brazil in 2013, the Ministry of Health (MOH) established the Safe Surgery Protocol and recommended the use of the surgical safety checklist as a tool help improving the care of individuals undergoing surgical procedures in different hospital settings around the country^4^. This checklist is a simple, easy to apply, and low-cost resource, used to identify, compare, facilitate the surgical team communication, and verify (check) a group of items and procedures during surgery to reduce failures in the process^3^ ^,^ ^4^. Studies show that this protocol is associated with better outcomes, with reduction in surgical complications and mortality^3,5 7^.

 The checklist is used in three moments, before anesthetic induction, before the surgical incision, and before the patient leaving the operating room. The application of this tool represents a strategy to assist the surgical team in the development of actions inherent to the perioperative period and to facilitate communication between the team, considering that the list is easy to understand and apply^3^. Although WHO has recommended its use, in Brazil the results of its implementation are diversified, and alternatives aimed at increasing adherence have been employed^8^. A systematic review and meta-analysis on the effects of the checklist use on reducing surgical complications observed that, despite the many studies available in the literature, many have methodological inconsistencies. In addition, the use of the checklist can reduce harm, but since adverse events are multifactorial, in the absence of consistent studies, one cannot consider these findings as definitive^6^. In developing countries, the use of this resource has shown better results, as well as stimulated reflections regarding safe practices in surgery^1^ ^,^ ^5^ ^,^ ^9^ ^,^ ^10^.

 Although some meta-analyses and systematic reviews did not clearly reveal good scientific evidence about the effects of using this tool^2^ ^,^ ^6^, it is another effective checking mechanism, all for the patient’s safety. Thus, surgical procedures are carried out with decreased postoperative complications, and soft skills are promoted, such as teamwork, leadership, and communication^10^ ^-^ ^12^. The lack of these factors can also be the cause of adverse events, as failures unrelated to the surgical technique are frequent and, therefore, can compromise patient safety^6^ ^,^ ^10^. However, there are few high quality, randomized clinical trials on the impact of the checklist on patient safety, which points to the need for robust studies that can support the perceived benefits^5^.

 To our knowledge, this is the first study developed in Brazil, in the neotropical climate region of the Midwest. To this end, we have established a joint proposal, aligned with the recommendations of the Safe Surgery Saves Lives Program^3^, with support from the managers of the County Health Department, Patient Security Centers (PSC), and nursing and surgery staff, providing an organizational climate favorable to implantation. With these results, we hope to encourage reflections and provide material that contributes to expand scientific knowledge and improve the care practice of operating room professionals regarding patient safety.

 Therefore, the goal of this study is to assess the responses of patients in each phase of the safe surgery checklist implementation process and its associated factors. We also verified the checklist use before and after the implementation of the protocol, through its presence in patients’ records.

## METHOD

### Study Scenario and Design

We conducted a cohort study, with assessment before and after the performed intervention, at the Southwest II Health Region, state of Goias, Brazil, with patients admitted for surgery in the operating rooms of public hospitals of small and medium size. We also carried out a retrospective analysis from the medical charts containing records of the checklist from elective and emergency procedures. The sample, both of patients and medical records, was non-probabilistic, selected consecutively throughout the collection period.

The realization of this study, part of the main project “Safe Surgeries Protocol in a Neotropical Region in Central Brazil”, presents results in two stages: before the implementation of the protocol of safe surgeries and after the protocol establishment process, based on WHO’s recommendations^3^ ^-^ ^4^. In Phase I, it comprised five municipalities^13^ (Aporé, Chapadão do Céu, Caiapônia, Mineiros, and Jataí), and in Phase II, four, as the Mineiros county was not initially assessed due to temporary renovations.

### Data Collection

**Figure 1:**
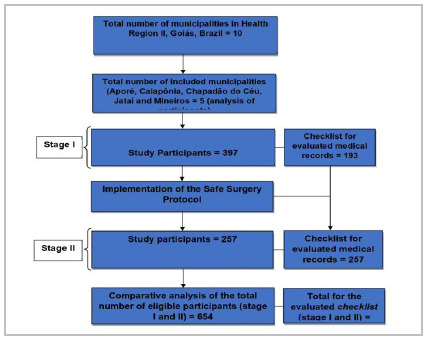
Flowchart of included study participants and evaluated medical records.

For the comparison of the checklist actions among the participants of the first and second phases, the study population consisted of patients undergoing elective or emergency surgery, during the period from June to December 2014 (Phase I), and from January to March 2016 (Phase II), as shown in the flowchart (Figure 1). Phase II was carried out one year after the beginning of the planning and implementation of the safe surgery protocol.

The main researcher immersed in the study field, aiming to expand incentive strategies and involvement of the patient safety culture, with the participation of surgeons, anesthetists, and administrative technicians from different sectors connected to the emergency room, together with the nursing team. The moments of continuing education were conducted by specialists in health surveillance and from the PSC, based on the WHO’s Safe Surgery Checklist^3^ and on the MOH’s Safe Surgery Protocol^4^. From this moment on, the safe surgery checklist was made available on all medical records of individuals undergoing elective and urgent operations in the health services.

Then, to collect data from the participants, we conducted an interview with a structured script, containing questions based on the items in the checklist that could be understood by the patient, covering the moments before anesthetic induction (four items out of six recommended) and before the surgical incision (two items out of five)^4^. We collected sociodemographic data (sex, age, education, income, and type of surgery), institutional information (hospital size, type of anesthesia, municipality of origin), and information from the checklist^3^ ^-^ ^4^ (confirmation of the patient’s name before and after entering the OR, confirmation concerning the procedure and the surgical site, signed consent for surgery and anesthesia, demarcation of the surgical site, introduction of team members, and confirmation of surgery).

The analysis included individuals 18 years old or older, undergoing elective or emergency operations, and able to communicate at the time of the interview. We excluded records with incomplete information. At the same time, we selected medical records of patients who underwent surgery for analysis as to the existence and completion of the checklist after its release.

### Inferential Analysis

In this study, we analyzed two sections from the database. We performed a bivariate analysis with the Chi square or Fisher exact tests to assess the proportion of responses from all patients of Phases I and II. To analyze the existence of the checklist in the records and its complete filing, we considered the outcome variable the existence or not of a filled safe surgery checklist. The variables with a p value < 0.20 in the bivariate analysis were tested in the multivariate analysis model. We estimated the association of a filled checklist with the independent variables using simple and adjusted Poisson Regression (PR), with respective 95% confidence intervals (95% CI). Thus, the variables tested in the model comprised surgeries performed in Phase I (mid-sized hospital) and the ones performed in Phase II: hospital size, sex, type of surgery, and antibiotic prophylaxis. We performed all analysis with the Statistical Package for the Social Sciences, SPSS version 23.0, IBM®, adopting the significance level of p < 0.05.

 The study is part of a larger project and was approved by the Ethics in Research Committee of the Federal University of Goiás, under protocol No. 37972114.6.0000.5083 / 2015. All participants signed an informed consent form.

## RESULTS

### Sociodemographic Data and checklist items

We interviewed 654 patients after their surgical procedures, 397 before implantation, in Phase I, and 257 in Phase II. Table 1 shows the sociodemographic data, before and after the training of professionals, respectively.

**Table 1:** Sociodemographic features and surgical items checklist from the responses of patients admitted to small and medium-sized hospitals, Southwest II Health Region, Goiás, Brazil.

Variables	Total n (%)	Patient N = 397 Phase I n (%)	Patient N = 257 Phase II n (%)	p*
Sex				0.001
Female	369 (56.4)	204 (51.4)	165 (64.2)	
Male	285 (43.6)	193 (48.6)	92 (35.8)	
Age				0.067
20-30	193 (29.5)	131 (33)	62 (24.1)	
31-40	226 (34.6)	128 (32.2)	98 (38.3)	
≥ 41	235 (35.9)	138 (34.8)	97 (37.7)	
Education				0.000
≤ 12 years of study	556(85.0)	358(90.2)	198(77)	
> 12 years of study	98 (15.0)	39 (9.8)	59 (23)	
Income (minimum wages)**				0.000
< 2	194 (29.7)	103 (25.9)	91 (35.4)	
2 - 5	402 (61.5)	245 (61.7)	157 (61.1)	
> 5	58 (8.90)	49 (7.50)	9 (3.50)	
Type of surgery				0.036
Elective	404 (61.8)	258 (65)	146 (56.8)	
Urgency	250 (38.2)	139 (35)	111 (43.2)	
Hospital size				0.273
Small	346 (53.0)	204 (51.4)	143 (55.6)	
Midium	307 (47.0)	193 (48.6)	114 (44.4)	
Type of anesthesia				0.908
General	120 (18.3)	72 (18.1)	48 (18.70 )	
Spinal	482 (73.7)	295 (74.3)	187 (72.8)	
Epidural	52 (8.00)	30 (7.60)	22 (8.60)	
Municipality of origin				0.000
Municipality of collection	366 (56.0)	249 (62.7)	117 (45.5)	
Another municipality	288 (44.0)	148 (37.3)	140 (54.5)	
Checklist items				
Confirmation of identification^1º^				0.000
Yes	217 (33.2)	97 (24.4)	120 (46.7)	
No	437 (66.8)	300 (75.6)	137 (53.3)	
Confirmation of surgical site^1º^				0.000
Yes	141 (21.6)	3 (0.80)	138 (53.7)	
No	513 (78.4)	394 (99.2)	119 (46.3)	
Consent for surgery and anesthesia^1º^				0.000
Yes	312 (47.7)	126 (31.7)	186 (72.4)	
No	342 (52.3)	271 (68.3)	71 (27.6)	
Surgical site demarcation^1º^				0.000
Yes	89 (13.6)	2 (0.50)	87 (33.9)	
No	565 (86.4)	395 (99.5)	170 (66.1)	
Introduction of team members^2º^				
Yes	167 (25.5)	v 5 (1.30)	162 (63.0)	
No	487 (74.5)	392 (98.7)	95 (37.0)	
Confirmation of surgery^2º^				
Yes	189 (28.9)	2 (0.50)	187 (72.8)	
No	465 (71.1)	395 (99.5)	70 (27.2)	

*Pearson Chi-square or Fisher exact test, p <0.05. **Minimum wage of R$ 724.00 in Phase I and R$ 788.00 in Phase II. ^1^Before anesthetic induction - first moment. ^2^Before surgical incision - second moment.

Of the 654 respondents, more than half were female (56.4%), aged 20 to 40 years (66%), with education up to 12 years of study (85%) and income between 2 and 5 minimum wages (61.5%). Among these, most underwent elective surgery (61.8%) and spinal anesthesia (73.7%). Most hospitals were small-sized (53%) and the participants lived in the same municipality (56%). There were statistically significant differences in patient’s responses between Phases I and II for sex (p = 0.001), education (p = 0.000), income (p = 0.000), type of surgery (p = 0.036), and municipality of origin (p = 0.000), (Table 1).

The bivariate analysis revealed significant associations (p <0.000) between patients’ responses and the six items assessed by the checklist (Table 2). Such results refer to the patients’ point of view, considering only the medium-sized hospital, in which the safe surgery checklist was already included in the records.

**Table 2:** Bivariate analysis of the responses of patients admitted to a medium-sized hospital, Southwest II Health Region, Goiás, Brazil, regarding the checklist items.

Checklist items	Phase I (N = 193)	Phase II (N = 114)	p*
	n (%)	n (%)	
Confirmation of identification^1º^			< 0.000
Yes	41 (21.1)	53(46.5)	
No	143 (78.8)	95 (53.5)	
Confirmation of surgical site^1º^			< 0.000
Yes	1 (0.50)	29 (25.4)	
No	192 (99.5)	85 (74.5)	
Consent for surgery and anesthesia^1º^			
Yes	67 (34.7)	64 (56.1)	
No	126 (65.3)	50 (43.9)	
Surgical site demarcation^1º^			
Yes	1 (0.50)	29 (25.4)	
No	192 (99.5)	85 (74.6)	
Introduction of team members^2º^			< 0.000
Yes	1 (0.50)	76 (66.7)	
No	192 (99.5)	38 (33.3)	
Confirmation of surgery^2º^			< 0.000
Yes	2 (1.00)	65 (57.0)	
No	191 (99.0)	49 (43.0)	

* Pearson Chi-square or Fisher exact test, p <0.05; ^*1*^ *Before anesthetic induction - first moment;* ^*2*^ *Before the surgical incision - second moment.*

Table 3 brings the analysis of the implantation process based on the surgery’s characteristics of the patients treated at the operating room. In six checklist items related to the time before the incision, there were significant differences (p < 0.001) between the patients’ responses regarding confirming the identification of the surgical site, the consent for surgery and anesthesia, the demarcated surgical site, the introduction of the team members, and the confirmation of the operation.

**Table 3:** Surgical characteristics (N = 450) regarding the existence of a safe surgery checklist in small and medium-sized hospitals, Southwest II Health Region, Goiás, Brazil.

	Checklist*			
Variables	Total n (%)	Yes n (%)	No n (%)	p**
Study phase				
Before training	193 (42.9)	135 (69.9)	58 (30.1)	
After training	257 (57.1)	248 (96.5)	9 (3.50)	
Hospital size				0.000
Small	143 (31.8)	134 (93.7)	9 (6.30)	
Medium	307 (68.2)	249 81.1)	58 (18.9)	
Sex				0.009
Male	203 (45.1)	163 (80.3)	40 (19.7)	
Female	247 (54.9)	220 (89.1)	27 (10.9)	
Age (years)				0.737
20-30	125 (27.8)	104 (83.2)	21 (16.8)	
31-40	163 (36.2)	141 (86.5)	22 (13.5)	
≥ 41	162 (36.0)	138 (85.2)	24 (14.8)	
Type of anesthesia				0.533
General	100 (22.2)	87 (87.0)	13 (13.0)	
Spinal	311 (69.1)	265 (85.2)	46 (14.8)	
Epidural	39 (8.7)	31 (79.5)	8 (20.5)	
Type of surgery				0.017
Elective	208 (46.2)	186 (89.4)	22 (10.6)	
Urgency	242 (53.8)	197 (81.4)	45 (18.6)	
Antibiotic prophylaxis				0.000
30-60 min before incision	225 (50)	206 (91.6)	19 (8.40)	
30 60 min after incision	207 (46)	165 (79.7)	42 (20.3)	
30 60 min after surgery	18 (4)	12 (66.7)	6 (33.3)	
Checklist completeness (N = 383)				
Completeness	Total	Phase I	Phase II	-
Complete	344 (89.8)	136 (39.5)	208 (60.5)	
Incomplete	39 (10.2)	30 (76.9)	9 (23.0)	-

* Pearson Chi-square or Fisher exact test, p <0.05.

### Presence of the checklist in the medical records and data completeness

Regarding the existence of the safe surgery checklist according to hospital size, Table 3 shows the results of 450 operations, corresponding to 193 (43%) operations carried out in the medium-sized hospital in Phase I, and 257 (57%) in Phase II. It also shows the distribution of the presence of the checklist regarding the lack of data related to the moments recommended in the checklist. It is noteworthy that the presence of the checklist in the medical records with complete data was greater in Phase II (96.5%) than in Phase I (69.9%), represented by the municipality that only provided the form in the medical record, but without having implemented the process by management, with training and involvement of the team. Thus, in Phase I, incomplete filling comprised about 76.9% of the checklist, decreasing to 23% in Phase II.

 The variables with statistical significance were subjected to multivariate regression analysis as to the adherence to the safe surgery checklist (existence of checklist) of the procedures (n = 450).

Table 4 shows the simple and adjusted regression analysis between adherence to the checklist (existence of the checklist in the medical record) and the independent variables. In the proposed model, the existence of the checklist was statistically associated with Phase II of the study, after the educational action (Prevalence Ratio [PR] 1.38, 95% CI: 1.25 1.51, p <0.000), medium-sized hospital (PR: 1.11, 95% CI: 1.0 1.17, p <0.00 0), sex (PR: 1.07, 95% CI: 1.0 1.14, p <0.010), type of surgery (PR: 1.07, 95% CI: 1.01 1.14, p <0.014), antibiotic prophylaxis 30 to 60 minutes before incision (PR: 1.10, 95% CI: 1.04 1.17, p <0.000), and antibiotic prophylaxis 30 to 60 minutes after the end of the incision (PR: 1.23, 95% CI: 1.04 1.45, p <0.015). In this same analysis, the existence of the safe surgery checklist was statistically associated with Phase II (adjusted Prevalence Ratio [aPR] 1.28, 95% CI: 1.01 1.62, p <0.034), in which the protocol implementation strategies took place in the institutions. 

**Table 4:** Multiple regression analysis of the safe surgery checklist (existence of the checklist) of operations (N = 450) in small and medium-sized hospitals, Southwest II Health Region, Goiás, Brazil.

Variables	PR (95% CI) #	p	aPR * (95% CI)	p
Operations carried out				
Before training	1		1	
After training	1.38 (1.25-1.51)	<0.000	1.28 (1.01-1.62)	0.034
Hospital size				
Small	1		1	
Medium	1.11 (1.0-1.17)	<0.000	0.89 (0.67-1.19)	0.459
Sex				
Female	1		1	
Male	1.07 (1.0-1.14)	0.010	1.03 (0.86-1.24)	0.683
Operation type				
Elective	1		1	
Urgency	1.07 (1.01-1.14)	0.014	1.04 (0.85-1.28)	0.647
Antibiotic prophylaxis				
30 60 min before incision	1		1	
30 60 min after incision	1.10 (1.04-1.17)	<0.000	1.03 (0.85-1.24)	0.761
30-60 min after surgery	1.23 (1.04-1.45)	0.015	1.10 (0.71-1.71)	0.640

# PR = Prevalence Ratio. CI - Confidence Interval. * aPR = Adjusted Prevalence Ratio.

## DISCUSSION

Brazil is a country with an extensive geographical area. However, there is national heterogeneity in the implementation of the safe surgery checklist in surgical centers, revealing different realities in the States of the Federation^14^ ^-^ ^16^. The State of Goiás, with an economy focused on agriculture and a neotropical climate, displays shortage of high-complexity health care centers in small and medium-sized cities. To our knowledge, this study is the first to present data on the use of this tool in Southwest Goiás, Midwest Brazil. It is, therefore, a contribution to public health, describing results of small and medium-sized hospitals, related to the patient’s perception about some of the checklist items. All the municipalities of the Southwest II Health Region are part of the neotropical region^13^, with distinct tropical climate characteristics. There are many challenges in public health and in perioperative care. During the study period, only one midsize municipality made the checklist available in the chart; however, the instrument was only attached to the record.

In this study, when analyzing the patient’s responses to the six checklist items, we identified differences of proportions in all the items between the first and second phases. Therefore, after the implementation and teams training, there was improvement in all evaluated aspects. We emphasize that the consent for surgery and anesthesia, which must be checked before anesthetic induction, raised questions and reflections about the process of communication between the team and the individual under care, as patients denied having been informed about the consent forms. However, when analyzing the medical records, we found forms attached to all records. The surgical environment comprises many details, nuances, techniques, and understanding of surgical times, requiring broad comprehension. Thus, understanding this inconsistency requires further studies, with appropriate methods. Nonetheless, this communication-related phenomenon also occurs in other scenarios, both in Brazil and in other countries^17^ ^-^ ^21^.

The use of the checklist is marked by common barriers and difficulties in hospitals in different countries, whether developed or developing. A research conducted in 33 Thai hospitals, based on content analysis, showed that the barriers are related to the lack of consistent policy, the existence of unqualified personnel, refusals, and resistance from the surgical team^19^. Thus, the checklist can be a potential instrument to improve team communication and to promote a safety culture, which can positively impact patients’ surgical care^12^. In this regard, a study carried out in Brasil, a strategy based on planning, doing and acting was used by the authors, in order to stimulate the increased use of the checklist by the leading doctors and the administrative team. Therefore, at the end of the study, there was a compliance rate of 89%^8^. 

Considering that the operating room is a complex, dynamic, and risky unit^22^, patient safety is a relevant factor for the implementation of strategies that minimize or prevent the occurrence of adverse events. In this regard, actions to improve the communication between professionals and patients are relevant and recommended by the Safe Surgery Saves Lives Program^3^. As for the consent for surgery and anesthesia, we observed that, in Phase I, more than half of patients (68.3%) referred not having signed it or received information about it, despite its presence in the records. It is thus necessary to reflect upon the communication processes in the different scenarios in the country. Corroborating these findings, both in Phases I and II, more than half of patients claimed that there was no confirmation of their identity.

However, a recent study held in one of the scenarios of this research showed that the identification of patients at the institution was not standardized, as verified by comparing the information inserted by professionals with the one provided by patients^22^.

Good practices that add value to the care quality in safety culture are important and, in this perspective, communication is part of them. We observed that the patients signed the consent form before the operation but did not remember having done so when approached soon after the procedure. We emphasize that this action should be performed even without the availability of the checklist, as recommended by the Ministry of Health, as a mandatory condition for the operation. After training, Phase II data showed a relative improvement, confirmed by 72.4% of participants stating they had been informed. A study conducted in a large hospital found that the signed informed consent form was present in 93.4% of records, however, the focus of that research is not presented from the patient’s perspective. The “patient identification, surgery, and surgical site” was the least checked item (85.8%)^16^.

 In Phase I of our study, only one medium-sized hospital made the checklist available on the chart. Even so, more than half of participants referred not having received information about, or not having noticed the performance of, the procedure confirmation. However, after training, the confirmation of the correct surgery with the patients improved, as well as the introduction of team members at the time of surgery. The National Health Vigilance Agency (ANVISA) Safe Surgery Protocol4 states that the person responsible for completing the checklist must confirm that the surgeon demarcated the surgery site on the patient’s body before anesthetic induction^4^; however, in studies carried out in Brazil, it appears we have different realities^1^ ^,^ ^7^ ^,^ ^9^ ^,^ ^14^.

The adoption of the recommendations of Safe Surgery Saves Lives Program shows growing concern of the institution and its professionals in the pursue of patients’ safety in the surgical context. In this sense, a study carried out in a large hospital in Brazil identified that the responsibility for conducting the safety check was of the room circulating nurse, with the participation of the anesthesiologist and the surgeon, since some items on the checklist are the responsibility of specific professionals^16^. Another study, conducted in Florianopolis with nurses in operating rooms, evaluated compliance with the ninth goal, which is to communicate effectively and to exchange information critical to the safe performance of the operation. There was an 84.5% participants’ compliance^23^. In a research conducted in Switzerland with surgeons and anesthesiologists to assess their opinion on the checklist, most agreed that this tool contributes to procedures’ safety and team communication; however, there is still resistance, both by some professionals^24^ and to its use^16^. 

We emphasize that only the checklist is not able to promote a safety culture in surgical care, given the complexity and the multiple facets of a surgical event^16^. On the other hand, it is necessary to reflect on the quality of studies published in the literature with different scenarios and methodological processes that hamper comparisons. A systematic review revealed a moderate quality of the studies, and some results were inconclusive as to reducing adverse events and mortality^25^. However, there are studies that have reported improvement in the rates of postoperative complications and mortality^2^ ^,^ ^6^.

 In the present study, the overall prevalence of the checklist in the medical records was higher (96.5%) in Phase II, after the implementation of the patient safety policy. The percentage of records without the checklist in the postoperative period dropped from 76.9% in Phase I to 23% in Phase II, proving that the use of the safety checklist has occurred in greater proportion. The use of the checklist in poor or developing countries, where the lack of resources and surgical practices do not yet contemplate new technologies existing in developed countries, tends to bring many benefits. These are associated with a reduction in mortality, dissemination of a patient safety culture, and a reduction in surgical site infections^10^ ^,^ ^19^ ^,^ ^26^. We highlight that, in the current research, in addition to the proportional increase in the use of the instrument, the completion of the fillings increased from 39.5% to 60.5%. In this regard, we infer that there was a positive response of the implementation of the safe surgery checklist in the public hospitals of the studied municipalities.

 A study performed in public, teaching hospitals in Brazil pointed out that there was a considerable decrease in the number of unfilled checklists, though with increasing number of incomplete instruments^1^. A research from Southern Brazil found no significant adhesion on the use of the instrument and the verification of the checklist items was not verbal^14^. The incomplete filling of the instrument is a phenomenon that can occur in other scenarios^1^ ^,^ ^15^ ^,^ ^17^ ^,^ ^20^. Different studies reported that the challenge of the checklist implementation continues, both in Brazil and worldwide, and suggestions aimed at better enforcement of the process involve professionals from all specialties^28^. As for the filling, it requires engagement of the operating room staff for greater adhesion^16^.

It is noteworthy that the experience with the use of the checklist is reported in many countries, and even then, adverse events related to surgical procedures may occur. The use of the checklist can contribute to the reduction of harm and fatal outcomes, despite the challenges arising from the frequent lack of data filling^16^ ^-^ ^21^.

This study contributes to the awareness about the use of the checklist in an economically important region of Brazil, with focus on agribusiness. The challenge remains of greater adherence to the checklist use by professionals at all stages, as well as of the promotion of continuing education in health services^27^. The use of the instrument requires a dynamic and co-participative intervention^28^, since in the opinion of nurses and surgeons, disagreements and conflicts still occur^27^ ^,^ ^30^. We emphasize that new technologies have been implemented, seeking to use the system through a computerized screen, in real time, which results in improved performance in the use of this tool^31^.

The limitations of this research were the type of study and the impossibility of establishing causality in a temporal sequence. The results refer to small and medium-sized hospitals, with inclusion of sample by convenience, with patients included in the study from the order of arrival at the operating room. Furthermore, we performed no sample calculation, which implies the impossibility of making inferences as to results` accuracy. Another limitation refers to the participants’ approach, assessing the first and second moments of the three recommended by WHO. These moments represent the patient’s greater susceptibility to surgical tension. With regards to retrospective data, due to the sample characteristics only the verification of the checklist items filling does not allow extrapolations about the effective conduction of the procedure.

However, these analyzes expand the local knowledge as for the use this tool under the patients’ point of view, regarding some of the checklist items. This study also showed that interventions with continued education, together with surgeons, nursing staff, anesthesiologists, and with the support from management and unit chiefs, add important contributions that may impact patients’ safety and improve team communication. Nevertheless, the lack of adequate filling of the checklist items and inconsistencies in responses as per the patients’ point of view demonstrated the need for greater involvement in the use of the checklist and for perioperative guidelines to patients. There is still need of support from managers, adding periodic evaluations to the process, and of feedback to the team about the impact of interventions.
